# Prevalence of primary and metastatic brain tumors: a five-year single-center survey in Zahedan (Iran)

**Published:** 2012-11

**Authors:** Mohammad Salehi, Abtin Shahlaee, Vafa Rahimi-Movaghar

**Affiliations:** ^a^Sina Trauma and Surgery Research Center, Department of Neurosurgery, Shariati Hospital, Tehran University of Medical Sciences, Tehran, Iran.; ^*b*^Research Centre for Neural Repair, University of Tehran, Tehran, Iran.

## Abstract

**Background:**

There are a few well documented reports about epidemiologic features of brain tumors (BTs) in Iran. The present study was aimed to epidemiologically review the prevalence and trends of brain tumors, treated in a neurosurgical center in Zahedan over five years.

**Methods:**

We analyzed the data compiled from 112 patients with brain tumors operated on in Khatam-ol-Anbia Hospital (Zahedan, Iran) during 1992 to 1996. Data related to the age, gender, tumor site, tumor pathology, symptoms, signs, and first clinical manifestation were collected and analyzed. The prevalence of different histological types of brain tumors was analyzed from the data of operative biopsies/ surgeries/ resections.

**Results:**

The male to female occurrence ratio was 1.07 (51.8% males, 48.2% females). Brain tumors were most commonly diagnosed in the 31-40 year age group (23.2%) followed by the 11-20 year (17.9%) and 51-60 year (15.2%) age groups. And finally, the 41-50 year age group showed the lowest percent (3.6%) of BTs. The five most common histological types of BTs were astrocytoma (25.9%) followed by meningioma (17.9%), pituitary adenoma (8.9%), oligodendroglioma (8.9%), and ependymoma (7.1%). 6.3% of tumors were of metastasis stage. We found that 26% of brain tumors were located in cerebellum, 26% in the parietal lobe, 13% in frontal lobe, and 10% in the temporal lobe. The most common primary symptoms were headache (40%), vomiting (13%), and seizure (10%). The most common overall signs/symptoms were headache (98%), vomiting (40%), hemiparesis (37%), papilledema (22%), and visual loss (20%).

**Conclusions:**

Findings of our study present an epidemiological basis for understanding the brain tumor burden in Iran. Moe comprehensice epidemiological studies of a prospective nature are further required in this respect.

**Keywords:**

Central Nervous System Tumors, Epidemiology, Brain tumors


					Volume 4, Suppl. 1 Nov 2012
Publisher: Kermanshah University of Medical Sciences
URL: http://www.jivresearch.org
Copyright:
Congress of Iranian Neurosurgeons, 14-16 November 2012, Kermanshah, Iran
Paper No. 3
Prevalence of primary and metastatic brain tumors: a five-
year single-center survey in Zahedan (Iran)
Mohammad Salehi a
, Abtin Shahlaee a
, Vafa Rahimi-Movaghar a,b,*
a
Sina Trauma and Surgery Research Center, Department of Neurosurgery, Shariati Hospital, Tehran University of Medical
Sciences, Tehran, Iran.
b
Research Centre for Neural Repair, University of Tehran, Tehran, Iran.
Abstract:
Background: There are a few well documented reports about epidemiologic features of brain tumors (BTs) in Iran.
The present study was aimed to epidemiologically review the prevalence and trends of brain tumors, treated in a
neurosurgical center in Zahedan over five years.
Methods: We analyzed the data compiled from 112 patients with brain tumors operated on in Khatam-ol-Anbia
Hospital (Zahedan, Iran) during 1992 to 1996. Data related to the age, gender, tumor site, tumor pathology,
symptoms, signs, and first clinical manifestation were collected and analyzed. The prevalence of different histological
types of brain tumors was analyzed from the data of operative biopsies/ surgeries/ resections.
Results: The male to female occurrence ratio was 1.07 (51.8% males, 48.2% females). Brain tumors were most
commonly diagnosed in the 31-40 year age group (23.2%) followed by the 11-20 year (17.9%) and 51-60 year
(15.2%) age groups. And finally, the 41-50 year age group showed the lowest percent (3.6%) of BTs. The five most
common histological types of BTs were astrocytoma (25.9%) followed by meningioma (17.9%), pituitary adenoma
(8.9%), oligodendroglioma (8.9%), and ependymoma (7.1%). 6.3% of tumors were of metastasis stage. We found
that 26% of brain tumors were located in cerebellum, 26% in the parietal lobe, 13% in frontal lobe, and 10% in the
temporal lobe. The most common primary symptoms were headache (40%), vomiting (13%), and seizure (10%). The
most common overall signs/symptoms were headache (98%), vomiting (40%), hemiparesis (37%), papilledema
(22%), and visual loss (20%).
Conclusions: Findings of our study present an epidemiological basis for understanding the brain tumor burden in
Iran. Moe comprehensice epidemiological studies of a prospective nature are further required in this respect.
Keywords:
Central Nervous System Tumors, Epidemiology, Brain tumors
* Corresponding Author at:
Vafa Rahimi-Movaghar: Associate professor of Neurosurgery, Research Deputy, Sina Trauma and Surgery Research Center, Sina Hospital,
Hassan-Abad Square, Imam Khomeini Ave, Tehran University of Medical Sciences, Tehran, Iran. Phone: (+98) 915 342 2682, (+98) 216 6757010 ,
Fax: (+98) 216 675 7009, Email: v_rahimi@sina.tums.ac.ir , v_rahimi@yahoo.com, (Rahimi-Movaghar V).

				

